# Monozygotic twin cases of endometriosis with Glanzmann thrombasthenia: a case report and review of literature

**DOI:** 10.1186/s13023-023-02694-6

**Published:** 2023-04-18

**Authors:** Samaneh Rokhgireh, Abolfazl Mehdizadehkashi, Shahla Chaichian, Mohammad Faranoush, Fardis Salmanpour, Noosha Samieefar, Roya Derakhshan

**Affiliations:** 1 Endometriosis Research Center, Iran University of Medical Sciences, Tehran, Iran; 2 Pediatric Growth and Development Research Center, Iran University of Medical Sciences, Tehran, Iran; 3 Student Research Committee, School of Medicine, Ahvaz Jundishapur University of Medical Sciences, Ahvaz, Iran; 4 USERN Office, Shahid Beheshti University of Medical Sciences, Tehran, Iran; 5 Network of Interdisciplinarity in Neonates and Infants (NINI), Universal Scientific Education and Research Network (USERN), Tehran, Iran

**Keywords:** Endometriosis, Glanzmann, Monozygotic twins

## Abstract

**Background:**

Glanzmann thrombasthenia (GT) is a rare bleeding disorder with a high prevalence in communities where consanguineous marriages are mainstream. Endometriosis is a chronic inflammatory disease, and its risk increases in women with menstrual periods of longer than six days. The phenotypic expression of endometriosis is determined by the frequency and rate of the menstrual flow, as well as genetic and environmental factors.

**Result and case presentation:**

14-year-old monozygotic twin sisters with GT who developed ovarian endometriosis were referred to Hazrat Rasoul Hospital due to severe dysmenorrhea. In ultrasonic examination, endometrioma cysts were reported in both patients. They both went under endometrioma cystectomy, and the bleeding was managed using antifibrinolytic drugs, followed by recombinant activated coagulation factor VII. Both were discharged after 3 days. In the ultrasound examination performed one year after the surgery, ovaries were normal in the first twin, while the second twin had a 28 × 30 hemorrhagic cyst in the left ovary.

**Discussion and conclusion:**

Menstrual bleeding and genetic factors are two theories that could be related to GT and endometriosis association, and GT could be considered a risk factor for endometriosis.

## Background

Glanzmann thrombasthenia (GT) is an autosomal recessive inherited platelet aggregation disorder caused by defects in the expression of platelet glycoprotein (GP) IIb/IIIa (integrin αIIbβ3), a platelet membrane receptor, suppressing platelet activation in response to agonists such as ADP, collagen, or thrombin [1, 2]. The prevalence of this rare inherited disorder is measured to be 1:1,000,000, while it is slightly prominent in women (58%) compared to men (42%) [3, 4]. Meanwhile, this value is estimated to be up to five times higher in the Middle East. It is also common among Palestinians, as well as in Iran, Iraq, Saudi Arabia, India, Jordan, and France, which could be attributed to the higher rate of consanguineous marriage in these areas. In Iran, GT’s prevalence is 1:200,000, having said that 86.6% of cases are reported in families with consanguineous marriages [5–7].

Endometriosis is defined as the presence of endometrial glands and stroma outside the uterine cavity, and it is known as a benign chronic inflammatory disease with a prevalence of 10% in women during their reproductive life. Pain (chronic pelvic pain, progressive dysmenorrhea, and dyspareunia) and infertility are two of the main symptoms for endometriosis. Laparoscopic excision of endometriosis is the main treatment for Endometriosis. [8, 9]. The US Center for Disease Control and Prevention (CDC) report has demonstrated that out of 217 women with inherited bleeding disorders, over half of the patients experienced dysmenorrhea, among whom endometriosis diagnosis was confirmed in 13% [10]. It has been hypothesized that GT itself may be a predisposing factor for endometriosis. Heavy menstrual bleeding and genetic factors are two theories which have been hypothesized to be related to GT and endometriosis, although not proven yet [11–13].

Glanzmann’s thrombasthenia pathology includes a prolonged bleeding time, absent or diminished clot retraction and absence of platelet aggregation in response to agonists such as ADP, collagen, thrombin and adrenaline, however, platelet aggregation is seen in the presence of ristocetin. Hence, surgical procedures has remained a significant challenge due to the probability of bleeding and a high incidence of alloimmunization due to repeated platelet transfusion [14]. The treatment of bleeding episodes in patients with GT who undergo scheduled surgery includes the management of acute bleeding and the prevention of bleeding complications during surgery. The choice of treatment depends on the severity of the bleeding, the availability of products, and the patient’s history of responses to treatment. It is recommended to use antifibrinolytic agents (such as aminocaproic and tranexamic acid), recombinant activated coagulation factor VII (rFVIIa), and platelet transfusion in such patients during surgery [11, 15].

This study aims to present 14-year-old monozygotic twin sisters with GT who further developed ovarian endometriosis to discuss the possible association of these two conditions. Furthermore, treatment through laparoscopic surgery and ways of managing bleeding-associated complications will be weighed up.

## Method

This was a case report study with a narrative review of previous studies of GT patients who developed endometriosis.

The two cases were 14-year-old monozygotic virgin twin sisters who were known cases of GT diagnosed at two months of age. The twins’ parents were related. The two sisters were simultaneously referred to Hazrat Rasoul Hospital of Iran University of Medical Sciences due to severe dysmenorrhea on 20 January 2021.

## Result

### Clinical history of the first twin

She was a virgin 14-year-old girl with a menarche age of 10 years (BMI: 29), a history of heavy menstrual blood loss, dysmenorrhea of 9/10 (visual analog scale of pain), without dyschezia, no menstrual-related urinary symptoms, and no history of hormonal diseases or surgery. She has had dysmenorrhea with a severity of 5/10 for the past three years, which has worsened over the past year. She was under treatment with Tranexamic Acid (250 mg) and Ferrosulfate and used recombinant factor VII in cases of severe menstrual bleeding. She had not received hormonal therapy or platelet transfusion to date. The blood group was O positive.

In a recent Doppler transrectal, trans-abdominal, and trans-labial ultrasound performed on 7 January 2021 due to severe dysmenorrhea, a 95-mm-sized endometrioma cyst with a focal echogenic pattern and a diameter of 46 mm was observed in the left ovary, suggesting a clot. No pelvic adhesion was seen in the sliding maneuver. Due to her unbearable pain, she was the first twin prepared for laparoscopic surgery.

### Clinical history of the second twin

She was a virgin 14-year-old girl with a menarche age of 10 years (BMI: 28.6), with the same presenting symptoms, signs and clinical history, and this twin also had been under the same treatment in severe menstrual bleeding episodes. he blood group was also O positive.

In the transrectal, trans-abdominal, and trans-labial ultrasounds performed on 7 January 2021, the right ovary showed an endometrioma cyst measuring 116 × 73 mm. There was no evidence of abnormal blood flow or cystic nodules in the Doppler ultrasound. The sliding maneuver revealed normal cervical motility with no evidence of pelvic adhesion.

### Surgical management

Due to more severe pain, the first twin was granted priority for laparoscopic surgery. Table 1 shows the preoperative lab results of both patients.

**Table 1:** Preoperative lab test of the twins

Lab test	First twin	Second twin
HB	12.7 g/dl	10.3 g/dl
PLT	260,000 per microliter	250,000 per microliter
Ferritin	58 µgr/l	56 µgr/l
AMH	0.75 ng/ml	0.56 ng/ml
CA125	32.63 U/ml	114.20 U/ml
HE4	27.2 pmol/ml	28.7 pmol/ml
ROMA	1.9%	2.4%

HB: Hemoglobin, PLT: Platelet Count, AMH: Anti-Mullerian Hormone, HE4: Human Epididymis Protein 4, ROMA: Risk of Malignancy Algorithm

#### The first twin surgery

As regards to obesity and lack of access to peripheral vessels, a right femoral central venous catheter was implanted by a vascular surgeon.

**Fig. 1:**
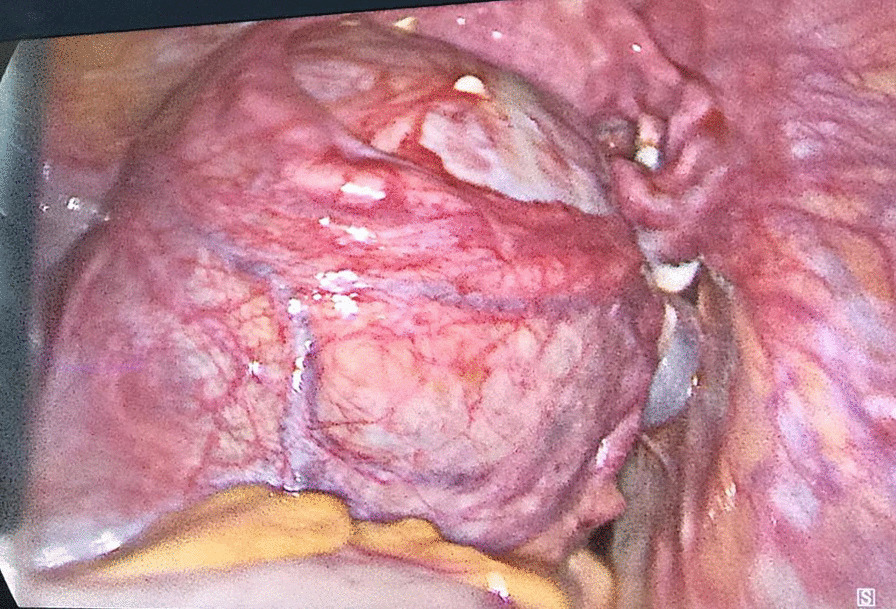
Laparoscopic image of the first twin

Half an hour before laparoscopy, NovoSeven (Recombinant factor VIIa, 90 µg/Kg) was infused intravenously. Two iso-group packed red cell units (checked for main and subgroup antibodies) and a single-donor platelet product were reserved and available. On laparoscopy, the first twin showed a normal cervix, while she had moderate tubular adhesion to the pelvic floor and both ovaries. A 3-cm para-tubular cyst was seen in the third distal part of the fallopian tube on both sides. The right ovary had adhesion to the fossa ovarica, which was released, and the left ovary contained a 10 × 10 cm cyst attached firmly to the fossa ovarica and the left uterosacral ligament (see Fig. 1). After hydrodissection, diluted vasopressin (20 units, a single injection) along with 200 mL normal saline (0.1 U/mL) were injected into the surface of the cyst from three different directions. Then cystectomy was performed, and the ovary was sutured. Endometriosis areas in the anterior clavicle, on both sides of the bladder, and in the right uterosacral ligament were ablated, and a nodule was removed from the left uterosacral ligament. In the middle of the surgery, a platelet concentrate was infused due to oozing. After ensuring a stable hemostatic condition, the Jackson drain was inserted. The surgery lasted 90 min.

Blood hemoglobin level was checked six hours after surgery, and considering the volume of bleeding in the drain, rFVII was infused on one occasion, and TXA (500 mg, four vials in 500 mL saline, slow drip) was infused every eight hours for three times. The patient was discharged on the third day after surgery (HB: 10.4).

#### The second twin surgery

**Fig. 2:**
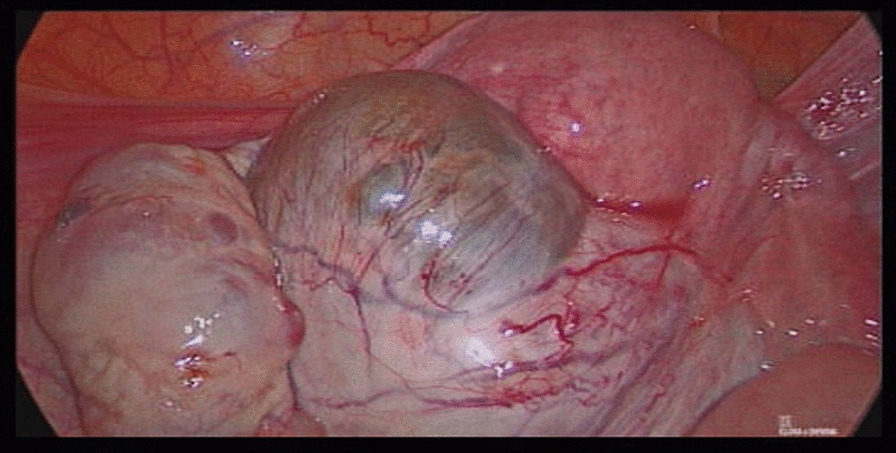
Laparoscopic image of the second twin

The second twin underwent laparoscopic surgery one week later. First, central femoral catheterization was performed on the right side. Half an hour before the surgery, adjusted for the patient’s weight, recombinant factor VIIa (NovoSeven, 90 µg/Kg) was infused intravenously. The endometrial cyst in the right ovary (size of 12 × 12 cm) was firmly attached to the dorsal uterus up to the uterus fundus and to the rectum in the back (see Fig. 2). Severe hydrosalpinx was observed in the right uterine tube, while the left side had mild hydrosalpinx. There were endometriosis areas on both sides of the bladder. Cystectomy was performed via hydrodissection using diluted vasopressin by the same method employed for the first twin. The ovary was released from the rectum and then sutured. The left uterosacral nodule was removed, and other endometriosis lesions were ablated. In the mid of the surgery, it was required to infuse platelets due to oozing. The surgery lasted 100 min. The patient received rFVII once six hours after the surgery, followed by the intravenous injection of TXA (500 mg, four vials in 500 mL saline, slow drip) every eight hours three times. The drain was extracted on the second day after the operation, and the patient was discharged (HB: 8.3) on the third day post-surgery (8.3).

### Follow-up

Both sisters were followed up at 3-month intervals and were prescribed Oral Contraceptive Pills (OCP). In the ultrasound examination performed one year after the surgery, ovaries were normal in the first twin, while the second twin had a 28 × 30 hemorrhagic cyst in the left ovary.

## Discussion

In this case report, we described two 14-year-old monozygotic twin sisters who were previously known as GT cases. They underwent laparoscopic surgery because of diagnosis of ovarian endometriosis, and were successfully managed.

Having said that heavy menstrual bleeding and genetic factors are theories which could relate GT and endometriosis [11]; Herein, we represented two cases that had both factors concomitantly. They were monozygotic twins which is the underlying genetic association, and they had episodes of heavy menstrual bleeding. Alatas et al. also reported two sisters endometriosis and Glanzmann’s thrombasthenia proposing that genetic factors and retrograde bleeding could associate these two conditions. Both cases were known cases of GT since childhood, and further developed Endometriosis [16].

As regards to the genetic side, many studies have suggested genetics as a principle factor for endometriosis. The disturbed regulation of a number of differentially expressed messenger RNAs in eutopic/ectopic endometrium by ovarian steroids may influence the expression of specific target genes and take part in the pathogenesis of endometriosis [17, 18]. By way of illustration, a study which was conducted on 3298 Australian monozygotic (MZ) and dizygotic (DZ) individuals to investigate the prevalence of twin pair concordance for endometriosis showed that genes could be a factor for endometriosis [14]. However,, more studies with larger sample and focus on tissue-specific biochemical and biological characterizations are needed to explain the genetic side of endometriosis [19].

The other aspect is related to bleeding, as some studies suggested that bleeding disorders could be a factor for endometriosis. Heavier menstruation which increases the amount of retrograde flow and symptomatic bleeding from extrauterine endometrial implants are explanation for this association [11]. To illustrate, one study proposed that the risk of endometriosis is higher in women with menstrual periods longer than six days which could support the one side which relates endometriosis to bleeding disorders [8]. According to a study by Poon et al., 98.2% of patients with GT manifest the clinical signs of heavy menstrual bleeding (HMB) [20]. Both our cases had the history of heavy menstrual bleeding episodes. As an example of another bleeding disorder, a study reported a prevalence of 30% for endometriosis among patients with Von Willebrand Disease compared to 13% in the control group [21].

The treatment of bleeding episodes in patients with GT who undergo scheduled surgery includes the management of acute bleeding and the prevention of bleeding complications during surgery. The choice of treatment depends on the severity of the bleeding, the availability of products, and the patient’s history of responses to treatment. It is recommended to use antifibrinolytic agents (such as aminocaproic and tranexamic acid), recombinant activated coagulation factor VII (rFVIIa), and platelet transfusion in such patients during surgery [15]. The most common treatment for bleeding in these patients has been the use of antifibrinolytic drugs (82%), followed by rFVIIa (42%), and our patients received the same therapy [22]. In a 35-year-old Omani woman reported by Pillaet al. who underwent laparotomy, the bleeding was also successfully managed with rFVIIa, platelet transfusion, and antifibrinolytics. [23].

Choosing a method of treatment of endometriosis is based on the knowledge of the disease and observations [24]. However, the gold standard of treatment for endomeriosis is laparoscopy. Here, we used hydro dissection via diluted vasopressin (20 units of Hypress®, Exir Pharmaceutical Co., Boroujerd, Iran, plus 200 mL of physiologic saline for 200-fold dilution, i.e., 0.1 U/mL) during endometrioma cystectomy in both patients, which reduced bleeding during the surgery.

Table 2. summarizes case reports of endometriosis in GT patients.

**Table 2:** Summary of GT cases with endometriosis

Authors	Year	Type of study	Age	Chief complaint	Medical history	Imaging		Treatment	Surgical findings	Preoperative management	Outcome
Alatas et al. [16]	2009	Case report (two sisters)	28 yrs	Primary infertility	- GT was diagnosed at the age of 3 - Frequent blood transfusion, mainly due to epistaxis - wedge resection for polycystic ovary syndrome -cystectomy for a presumed left ovarian chocolate cyst 2005	Transvaginal ultrasonography demonstrated a 3.5 cm cystic lesion suggestive of endometrioma		Surgical exploration- laparoscopy- cystectomy	- Diffuse adhesions - superficial endometriotic lesions over the left ovarian fossa - a right ovarian cyst	- Four units of apheresis platelet concentrate - one unit of whole blood	Discharged after 3 days
			24 yrs	Pelvic mass discovered following abdominal pain and distension for 2 years and	- GT was diagnosed at the age of 11 following by gastrointestinal tract bleeding - History of Hepatitis C	MRI showed a huge, septated, cystic mass (extending from the pelvic floor to the upper abdomen)		Surgical exploration- laparotomy- cystectomy and partial omentectomy	- A cystic mass of about 20*15 cm, firmly attached to the adjacent tissues - Focal necrotic areas in Omentum surrounding the mass - the mass composed of two separate cysts bilaterally originating from ovaries	Not mentioned	Discharged after 5 days
Imperiale et al. [11]	2015	Letter to the editor (three sister, of whom two were twins)	28 yrs	Dysmenorrhea, deep dyspareunia and severe menometrorrhagia	- GT was diagnosed after severe epistaxis a few days after birth	Transvaginal pelvic ultrasounds demonstrated a 70-mm hypoechoic and corpuscular cystic mass suggestive of endometrioma and a suspected intrauterine polyp of 20 mm (left adnexal)	After 11 months of medical follow-up to avoid surgery, transvaginal ultrasound (TVUS) revealed a new endometriotic cyst of 34 × 34 mm in left ovary	Surgical exploration- laparoscopy -hysteroscopic polyp removal	-	- 3 months of gonadotropin-releasing hormone analogs (GnRH-a) (triptorelin acetate 3.75 mg, intramuscular once a month) prior to surgery - rFVIIa (~ 90 mcg/kg) before and after surgery - Tranexamic acid 500 mg during the perioperative period	Postoperative TVUS showed the presence of a hematometra of 11.7 mm which was resolved and after 30 days patient was discharged
			40 yrs	Severe menorrhagia, mild dysmenorrhea and deep dyspareuni	- GT - severe heavy menstruation after the menarche	Abdominal and vaginal ultrasonographywas consistant with physical examination that endometriotic nodule of about 50 mm in diameter in the rectovaginal septum was recognized		Medical treatment and follow-up	-	-	Follow-ups were satisfying
			28 yrs	Heavy menstrual bleeding	- GT	Transvaginal ultrasonography showed a 35 mm cystic lesion with mixed echogenicity in the right ovary, not vascularized at Doppler		Medical treatment and follow-up	-	-	Follow-ups were satisfying
Pillai et al. [23]	2019	Case report	35 yrs	Infertility	- GT was diagnosed since childhood following episodes of epistaxis and heavy menstrual bleeding	Pelvic MRI demonstrated a 6,4 cm left-ovarian cyst, suggestive of endometrioma		Surgical exploration- laparotomy- ovarian cystectomy- intraperitoneal-drain		- rFVIIa 90 lg/kg intra- venously - One unit of packed red cells - Three units of platelet transfu- Sion -Tranexamic acid every 1 g 6 h	Discharged after 6 days

## Conclusion

Heavy bleeding along with the genetic factors might make a person susceptible to endometriosis, although not proven yet. Our study is similar to the previous studies that support the association. Likewise, endometriosis higher prevalence among women with hemorrhagic diseases require further studies in these high-risk groups. Further evaluations are recommended to find the genes involved.

Severe menstrual bleeding, frequent hospitalizations, and blood transfusions are common events in GT patients which could affect these patients quality of life [20]. Therefore, it is vitally important to develop strategies of bleeding management, particularly in surgeries with high risk of bleeding. We highly recommend to conduct further studies in these patients, and develop therapeutic strategies and guidelines to manage bleeding episodes while surgery.

Considering the relatively high prevalence of GT in Iran and the lack of sufficient data on the incidence of endometriosis in people with this genetic disorder, it is recommended to develop a specific therapeutic strategy for these patients. Generally, such patients can undergo minimally invasive surgeries only after preparing the patient and providing necessary blood products according to patient condition, such as rFVIIa and platelet concentrates.

## Data Availability

Not applicable.

## Abbreviations

GT: Glanzmann thrombasthenia
GP: Platelet glycoprotein
ADP: Adenosine diphosphate
CDC: Center for Disease Control and Prevention
rFVIIa: Recombinant activated coagulation factor VII
BMI: Body Mass Index
TXA: Tranexamic acid
HB: Hemoglobin
OCP: Oral contraceptive pill
MZ: Monozygotic
DZ: Dizygotic
HMB: Heavy menstrual bleeding
